# Impact of visual stimulus complexity on associative learning and associated reaction times in migraine patients

**DOI:** 10.1038/s41598-025-98187-6

**Published:** 2025-04-22

**Authors:** Kálmán Tót, Gabriella Eördegh, Noémi Harcsa-Pintér, Adél Papp, Balázs Bodosi, Gábor Braunitzer, János Tajti, Anett Csáti, Attila Nagy

**Affiliations:** 1 Department of Physiology, Albert Szent-Györgyi Medical School, University of Szeged, Dóm tér 10, Szeged, 6720 Hungary; 2 Department of Theoretical Health Sciences and Health Management, Faculty of Health Sciences and Social Studies, University of Szeged, Szeged, Hungary; 3 Sztárai Institute, University of Tokaj, Sárospatak, Hungary; 4 Department of Neurology, Albert Szent-Györgyi Medical School, University of Szeged, Szeged, Hungary

**Keywords:** Human, Associative equivalence learning, Cognitive functions, Neurology, Memory, Migraine, Migraine, Learning and memory

## Abstract

The semantic complexity and verbalizability of visual stimuli can influence associative learning. The Rutgers Acquired Equivalence Test (RAET) uses semantically rich stimuli (faces and colored fish) to assess associative learning and generalization, while a modified version, the Polygon test, employs simpler stimuli with reduced semantic content (grayscale circles and geometric shapes). Although cognitive alterations are well-documented in migraine patients during interictal periods, the impact of visual stimulus complexity on associative learning and reaction times has not been studied. Forty-one migraine patients without aura completed both the RAET and Polygon tests. Performance metrics included acquisition error ratios, retrieval and generalization error ratios, and reaction times. The two tests were compared using non-parametric statistical methods. Migraine patients demonstrated comparable acquisition performance on the RAET and Polygon test. However, reaction times were significantly longer in the Polygon test across both acquisition and test phases. Retrieval and generalization performance were also similar between tests, despite longer reaction times with semantically reduced stimuli. Migraine patients showed consistent learning performance across visual stimuli of varying semantic complexity. Prolonged reaction times with simpler stimuli suggest increased cognitive demands, potentially mitigated by cortical compensatory mechanisms that maintain learning ability under challenging conditions.

## Introduction

Several studies have reported that migraine patients may experience cognitive deficits not only prior to migraine attacks but also during the interictal period^1–4^. A meta-analysis revealed a significant negative impact of migraine during the interictal period on complex attention, executive function, memory, and spatial cognition^5^. Alterations in visual processing have also been documented, ranging from basic visual functions such as contrast sensitivity to more complex tasks like visually guided acquired equivalence learning^6–12^.

Equivalence learning is a form of associative learning in which equivalence between two or more stimuli is formed if they share common consequences. Additionally, this equivalence can be generalized to new instances if a new consequent appears^13,14^. To study visually guided equivalence learning, Myers and colleagues^15^ developed a computer-based cognitive test known as the Rutgers Acquired Equivalence Test (RAET). The task involves learning associations between visual stimulus pairs through trial-and-error learning. Some stimuli serve as antecedents (different drawn schematic faces), and others as consequents (different colored fish). The test consists of two main phases: the acquisition phase and the test phase.

In the acquisition phase, participants gradually learn the associations based on feedback provided by the software. In the test phase, these associations are evaluated. Additionally, new consequents, not presented or taught during the acquisition phase, are introduced. These new consequents can be linked to a given antecedent without additional feedback, relying on predictable associations from the previously learned ones (transfer or generalization). Variations of the test with different visual stimuli have also been used in other studies^14,16,17^.

The primary advantage of this test is that it engages distinct neural structures during its two phases. The basal ganglia–frontal cortex loops play a critical role in the acquisition phase, while the hippocampal region is essential during the test phase. This has been demonstrated through studies involving specific patient populations and neuroimaging techniques^14–16,18^. Multiple studies have shown that the hippocampi are both morphologically and functionally altered in migraine, which could potentially indicate cognitive dysfunction^19–21^. Given that the hippocampus plays a critical role in the test phase of RAET, such alterations may impact associative learning performance in migraine patients. Additionally, while some studies suggest basal ganglia involvement in migraine, its role remains less well characterized^22–24^.

The stimuli used in the RAET (faces and fish) are complex, detailed, easy to verbalize, and possess high semantic content (e.g., gender, age, hair color), which can influence the learning process. Studies have shown that more verbalizable stimuli positively affect visual memory performance and category learning^25,26^. According to a learning model in category learning, implicit and explicit memory systems contribute to learning simultaneously but competitively. When a rule can be verbalized during the learning process, learning occurs explicitly^27,28^.

Furthermore, the semantic content of stimuli may be semantically congruent and related to preexisting knowledge (conceptual relatedness) and prior experiences, which can enhance the learning process and memory formation^29–31^. A recent study suggests that conceptual relatedness may also facilitate generalization in an acquired equivalence task^17^.

The number of features can also influence learning^32–35^. For faces in the RAET, this involves many features (e.g., face shape, size of the eyes, nose, and mouth, color and length of the hair). In contrast, the polygons used in our study have significantly fewer features (e.g., the angles and lengths of the polygon sides).

Considering this, we aimed to reduce the complexity (semantic content and verbalizability) of the visual antecedent and consequent stimuli. To this end, we developed a new equivalence learning test with the same structure as the original RAET but employing reduced visual stimuli (polygons)^36^. In this new test, called the Polygon test, grayscale circles were used as antecedents, and different two-dimensional geometric shapes served as consequents. In a previous study involving a healthy adult population, we found that acquisition phase performance was significantly worse in the Polygon test, but retrieval and generalization remained unaffected^36^.

One possible explanation is that the Polygon test relies more heavily on implicit learning, requiring more trials and time to establish associations^37,38^. If this is the case, it may be more sensitive to the functioning of the basal ganglia–frontal cortex loops. As the Polygon test is hypothesized to rely more on basal ganglia-frontal cortex loops, it is important to explore whether migraine patients show a differential performance pattern across RAET and Polygon tasks compared to healthy individuals.

Previous studies have investigated associative learning in migraine patients using the RAET paradigm. Őze et al.^11^ examined learning in healthy and migraine adults using a modified RAET with high semantic content stimuli. These finding suggested some weak differences in acquisition and robust differences in generalization. Giricz and colleagues^9^ expanded on this by comparing four groups (healthy and migraine adults and children), revealing developmental differences. Tót and colleagues^39^ introduced a multisensory RAET, demonstrating enhanced multimodal integration in adult migraine patients.

Unlike these studies, our research investigates performance differences within the same migraine population across two paradigms: RAET (high semantic content) and Polygon (low semantic content). This approach aligns with the findings of Eördegh et al.^36^, who observed that healthy adults faced greater challenges during the acquisition phase of the Polygon test compared to the RAET, while retrieval and generalization remained unaffected. Our goal was to determine whether migraine patients exhibit a similar pattern or if their cognitive strategy differs, thereby providing insight into how stimulus complexity and verbalizability influence migraine-associated learning.

Given the well-documented hippocampal dysfunction in migraine, we hypothesize that migraine patients may show impairments in the test phase of RAET, which relies on hippocampal function^11^. Additionally, if the basal ganglia–frontal cortex loops are affected in migraine, we may observe differences in acquisition phase performance, particularly in the Polygon test, which is thought to engage this circuit more heavily.

## Results

In the present study, we analyzed data from 41 migraine patients. To investigate potential practice effects, we divided participants into two groups based on which test was presented first and conducted a Mann–Whitney U test to compare performance between these groups.

The results showed no significant differences for most measures, except for RAET acquisition error ratio (U = 127.5, *p* = 0.0376) and Polygon acquisition reaction times (U = 111.0, *p* = 0.0121). These isolated significant differences may indicate minor learning effects, but given the overall lack of systematic differences, practice effects are unlikely to have influenced the main findings.

### Acquisition phase

**Fig. 1:**
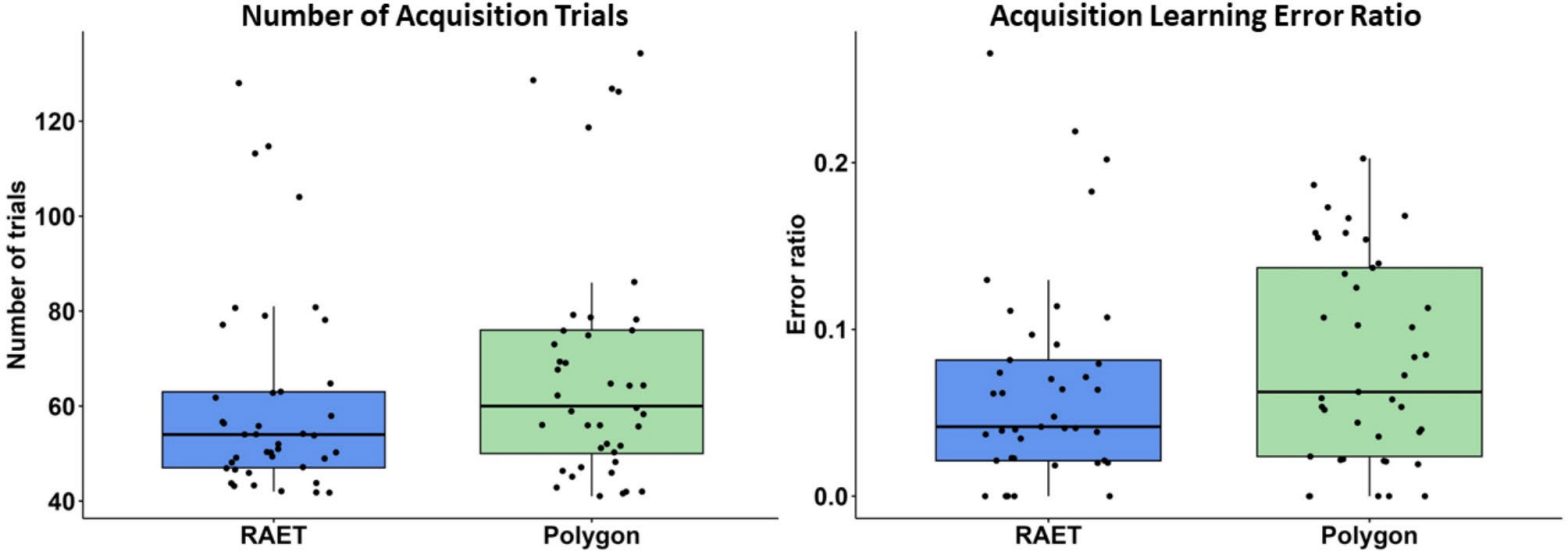
Performance in the acquisition phase. The upper margin of the box represents the third quartile (75th percentile), and the lower margin represents the first quartile (25th percentile). The line within the box indicates the median (50th percentile). Whiskers extend to the smallest and largest data points within 1.5 times the interquartile range (IQR) from the first and third quartiles, respectively. Each point corresponds to an individual data point, and points beyond the whiskers are considered outliers.

Although the migraine patients required more trials to learn the associations in the Polygon test compared to RAET, this difference was not statistically significant between the two tests in the number of acquisition trials (NAT) required to learn the associations (Z = 1.361, *p* = 0.174, *r* = 0.218, η^2^ = 0,047, power = 0.366). The median NAT in the RAET was 54 (Q1: 47–Q3: 63), and in the Polygon test, it was 60 (Q1: 50–Q3: 76) (Fig. 1).

The acquisition learning error ratios (ALER, calculated by dividing the number of incorrect answers by the total number of acquisition trials) showed a similar trend but were also not significantly different between the RAET and the Polygon test (Z = 1.102, *p* = 0.270, *r* = 0.176, η^2^ = 0,031, power = 0.259). The median ALER in the RAET was 0.042 (Q1: 0.021–Q3: 0.082), and in the Polygon test, it was 0.063 (Q1: 0.024–Q3: 0.137) (Fig. 1).

**Fig. 2:**
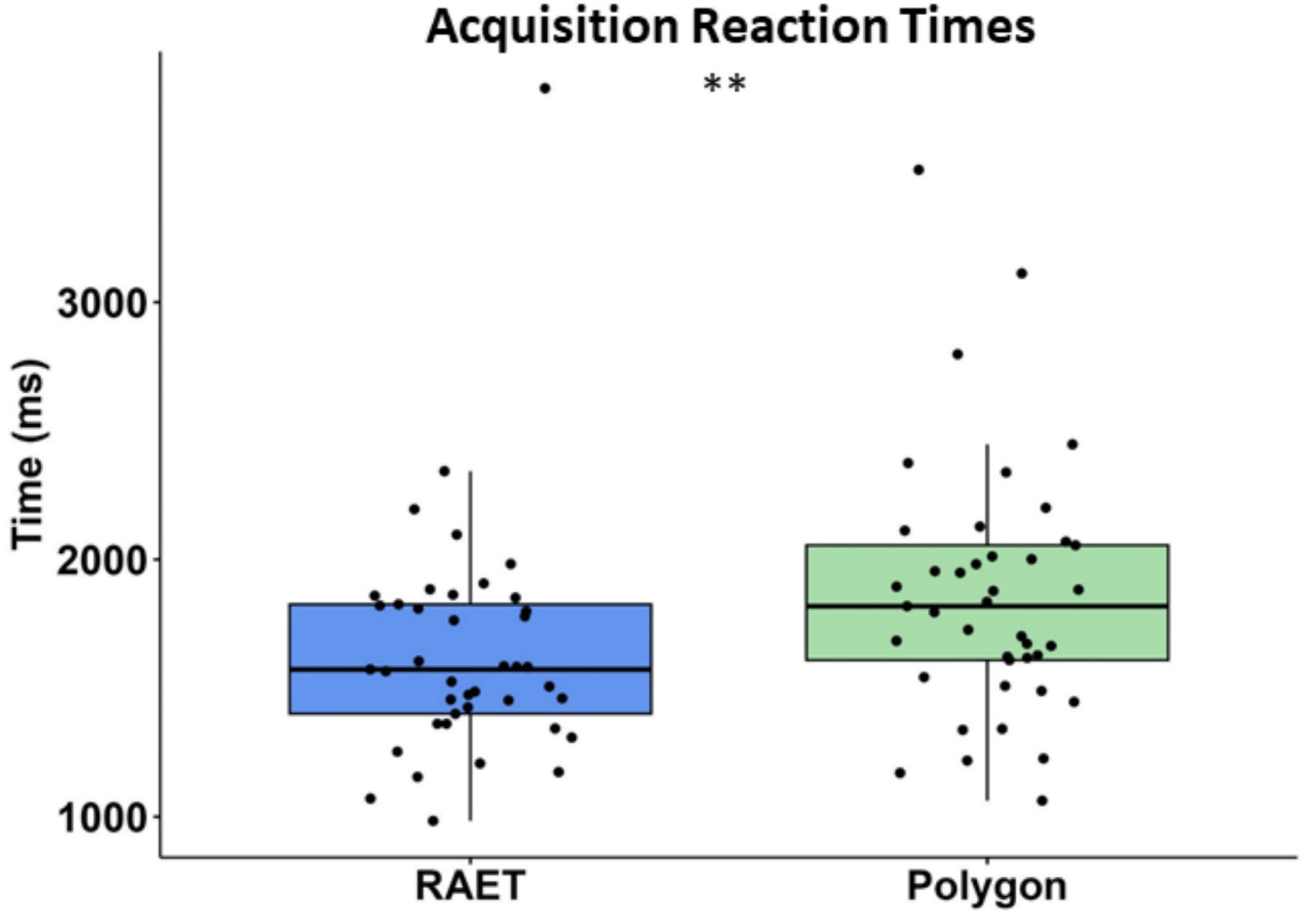
Reaction times in the acquisition phase. The two asterisks (**) indicate a significant difference at the level of *p* < 0.001. All other conventions are the same as in Fig. 1.

The reaction times (the time between the appearance of the stimuli and the participant’s response) for the acquisition trials (ART) were significantly longer in the Polygon test (Z = 2.663, *p* = 0.008, *r* = 0.416, η^2^ = 0,173, power = 0.887). The median ART in the RAET was 1573 ms (Q1: 1400 ms–Q3: 1826 ms), while in the Polygon test, it was 1818 ms (Q1: 1609 ms–Q3: 2055 ms) (Fig. 2).

### Test phase

**Fig. 3:**
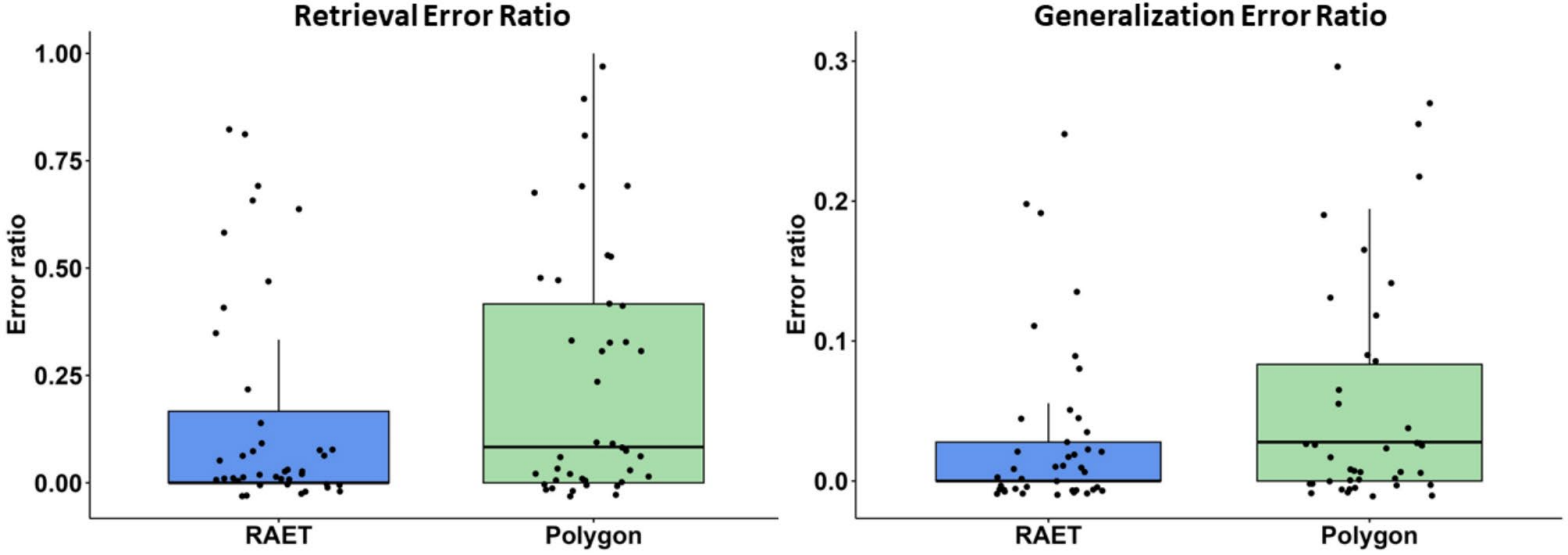
Performance in the test phase. All conventions are the same as in Fig. 1.

There was no significant difference in the retrieval error ratios (RER, calculated by dividing the number of incorrect answers by the total number of retrieval trials) between the two tests (Z = 1.177, *p* = 0.239, *r* = 0.227, η^2^ = 0,051, power = 0.282). The median RER in the RAET was 0.00 (Q1: 0.00–Q3: 0.028), and in the Polygon test, it was 0.028 (Q1: 0.00–Q3: 0.083). Similarly, no significant difference was found in the generalization error ratios (GER, calculated by dividing the number of incorrect answers by the total number of generalization trials) between the tests (Z = 1.373, *p* = 0.170, *r* = 0.255, η^2^ = 0,065, power = 0.367). The median GER in the RAET was 0.00 (Q1: 0.00–Q3: 0.167), while in the Polygon test, it was 0.083 (Q1: 0.00–Q3: 0.417) (Fig. 3).

**Fig. 4:**
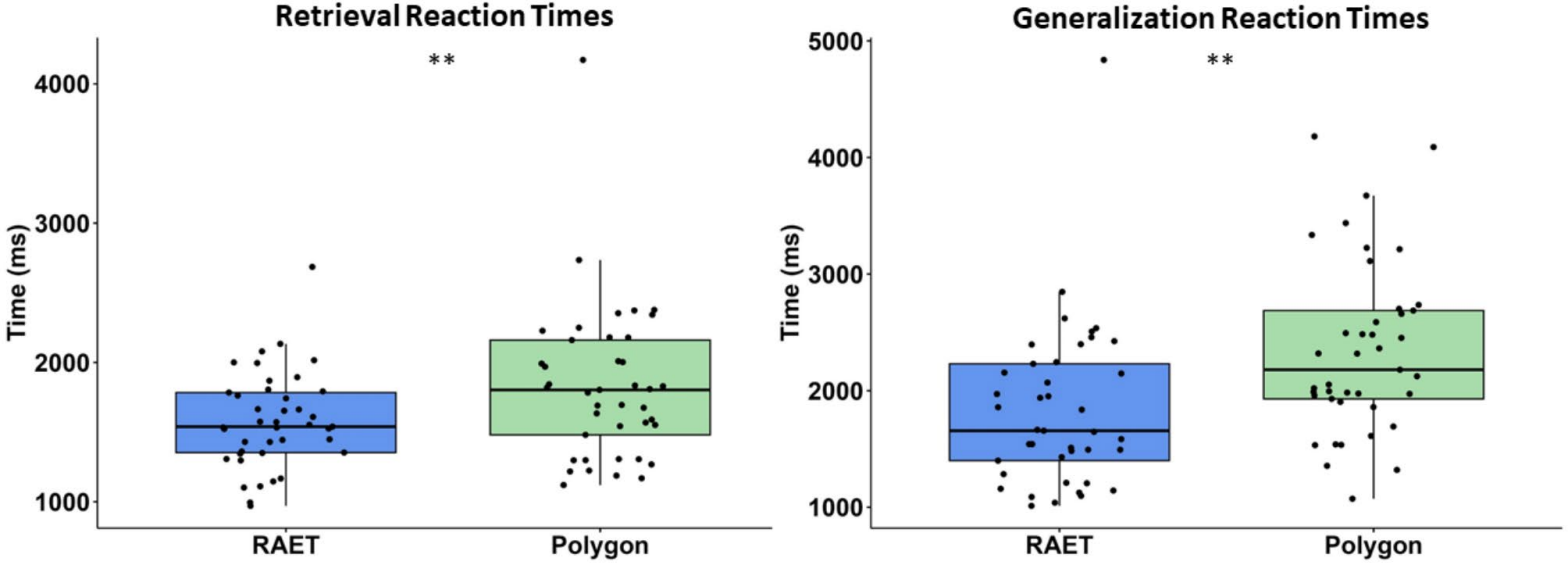
Reaction times in the test phase. All conventions are the same as in Figs. 1 and 2.

In contrast to performance measures, reaction times differed significantly between the two tests. Longer reaction times were observed in the Polygon test for both retrieval (RRT; Z = 2.819, *p* = 0.005, *r* = 0.440, η^2^ = 0,194, power = 0.918) and generalization (GRT; Z = 3.078, *p* = 0.002, *r* = 0.481, η^2^ = 0,231, power = 0.956). The median RRT in the RAET was 1540 ms (Q1: 1353 ms–Q3: 1784 ms), while in the Polygon test, it was 1803 ms (Q1: 1480 ms–Q3: 2161 ms). The median GRT in the RAET was 1657 ms (Q1: 1400 ms–Q3: 2231 ms), compared to 2180 ms (Q1: 1930 ms–Q3: 2687 ms) in the Polygon test (Fig. 4).

Table 1 presents the descriptive statistics for the RAET and polygon tests, including the median values along with the first (Q1) and third (Q3) quartiles. These values illustrate the distribution of performance across the two conditions, highlighting differences in response times and accuracy measures between the two test paradigms.

**Table 1:** Descriptive statistics of the performance in the RAET and polygon tests.

Variable	RAET			Polygon		
	Median	Q1	Q3	Median	Q1	Q3
NAT	54	47	63	60	50	76
ALER	0.042	0.021	0.082	0.063	0.024	0.137
ART	1573 ms	1400 ms	1826 ms	1818 ms	1609 ms	2055 ms
RER	0.00	0.00	0.028	0.028	0.00	0.083
GER	0.00	0.00	0.167	0.083	0.00	0.417
RRT	1540 ms	1353 ms	1784 ms	1803 ms	1480 ms	2161 ms
GRT	1657 ms	1400 ms	2231 ms	2180 ms	1930 ms	2687 ms

### Comparison of learning performances of migraine patients with those of the previously collected control population

To further examine differences between migraine patients and healthy controls from our previous study^36^, we conducted Mann–Whitney U tests comparing seven performance parameters (NAT, ALER, ART, RER, RRT, GER, and GRT) across the RAET and polygon tests.

The results revealed no significant differences between migraine patient and healthy control groups for most parameters in either test (*p* > 0.05). However, in the polygon test, a significant difference was observed in GER (U = 860.5, *p* = 0.047), with the healthy group exhibiting slightly better performance. In the RAET test, two parameters showed significant differences: RRT (U = 726.0, *p* = 0.004) and GRT (U = 618.0, *p* = 0.002), where the migraine group outperformed the healthy controls.

## Discussion

To our knowledge, this is the first study to investigate how the semantic content and complexity of visual stimuli affect associative learning and related memory processes in an acquired equivalence learning task among migraine patients. For this purpose, we applied two tests differing in the semantic content and, consequently, the complexity of the visual stimuli. In the RAET, the stimuli were drawn faces and fish, while in the Polygon test, they were grayscale two-dimensional geometric shapes. We argue that geometric shapes have lower semantic content and are therefore less complex compared to the colorful, detailed drawn faces and fish^32–35^. In the present study, we found no significant differences in performance between the two tests. However, reaction times were significantly longer in the Polygon test.

In our previous study^36^, which compared the same two tests in a healthy adult population, we found that learning performance was significantly worse in the Polygon test, while retrieval and generalization were unaffected. We hypothesized that this was because geometric shapes have lower semantic content and are less verbalizable, making learning more likely to occur implicitly. Consequently, significantly more trials and time were required to learn the associations, and error ratios were significantly higher during the acquisition phase.

In the present study, we observed a similar tendency in the acquisition phase among migraine patients, although the effect was less pronounced than in the healthy population. Migraine patients required more trials to learn the associations in the Polygon test, and their error ratios were higher, but these differences were not statistically significant.

One possible explanation is that migraine patients may engage in cognitive adaptations that help maintain learning performance. Previous research has suggested that individuals with migraine may develop heightened capabilities in specific cognitive domains, possibly as a response to chronic neurological challenges. For instance, our earlier study^39^ found that migraine patients outperformed healthy controls during the acquisition phase of an audiovisual equivalence learning test (SoundFace), based on the original RAET. Additionally, a large-scale study involving over a thousand participants found that migraine patients scored higher than controls on the Mini Mental State Examination, a test assessing executive functions and fine motor skills^40^.

This compensation may involve the basal ganglia–frontal cortex loops, which are known to play a crucial role in implicit learning. Evidence suggests that brain structures associated with declarative memory can compensate for basal ganglia dysfunctions in certain psychiatric conditions, including specific language impairments, dyslexia, autism, obsessive-compulsive disorder (OCD), and Tourette syndrome^41^. Both OCD and Tourette syndrome have been linked to basal ganglia impairments^42–44^. Neuroimaging studies investigating procedural learning have shown no significant behavioral differences between OCD patients and healthy individuals. However, OCD patients exhibited reduced activation in basal ganglia circuits and increased activity in brain areas associated with declarative memory^45–47^. This has led researchers to suggest that OCD patients may rely on declarative memory as a compensatory strategy for tasks that typically depend on procedural learning.

Previous neuroimaging findings indicate that migraine is associated with structural and functional alterations in the hippocampus, a region implicated in both declarative and procedural learning. Some researchers have suggested that these changes might influence memory processing and learning strategies^11,19–21^. While we did not directly measure neural mechanisms in the present study, these findings suggest that a similar process may be at play in migraine patients, potentially influencing their learning performance. Further support for this idea comes from Wen et al.^40^, who found that migraine patients performed better on the Purdue Pegboard Task, a psychomotor test commonly used to assess basal ganglia function, hinting at the possibility of enhanced implicit learning processes.

In a neuroimaging study, migraine patients demonstrated maintained performance on a visuospatial task (angle discrimination test) compared to the control group^49^. This superior performance was associated with increased activation of the right insula and orbitofrontal cortex. The authors suggested that these findings may indicate a compensatory mechanism involving brain areas commonly associated with nociception. However, the orbitofrontal cortex is also connected to the basal ganglia, a circuit involved in various cognitive and associative functions^43,50^.

In the test phase, no significant differences were found between the two tests in either retrieval or generalization. A similar tendency was observed in healthy individuals^36^. However, reaction times were significantly longer in the Polygon test for both retrieval and generalization. This pronounced difference was not observed in healthy individuals^36^, suggesting that these processes may be more demanding for migraine patients.

For more insights we compared the current data of migraine patients with a healthy controls from our previous study^36^. No significant differences were found in most performance parameters, except three (generalization error ratio in the Polygon test; and retrieval and generalization reaction times in RAET). However, the two groups were not matched for age, gender, and education, this comparison should be interpreted with caution. These results also highlight the need for future studies to include a directly-matched healthy control group to strengthen these findings.

This study has several other limitations that should be considered when interpreting the findings. Although our sample size of 41 participants was sufficient to detect larger effects, it may still limit the generalizability of the results, particularly for smaller effects. Replication with an independent and larger participant group is needed to validate these conclusions and ensure broader applicability. Furthermore, the unequal gender distribution may have influenced our results. Women with migraine often experience more frequent, longer-lasting, and more severe headaches compared to men^51^, which may lead to more pronounced cognitive impairment. This could, in turn, enhance the suggested compensatory mechanisms. Moreover, we acknowledge that our comparison to a previous study with a more balanced gender distribution^36^ may be influenced by potential sex differences in cognitive learning and performance^52^. Additionally, the study relies solely on behavioral data and insights from previous research; neuroimaging investigations are required to provide direct evidence of the neural mechanisms underlying the observed phenomena. Lastly, follow-up assessments of patients were not conducted after the testing session, raising the possibility that preictal measurements could have influenced the results.

Based on our results, we conclude that visually guided equivalence learning in migraine patients is influenced by the complexity of the visual stimuli, although this effect appears to be less pronounced than in healthy individuals. Our findings raise the possibility that alternative cognitive processing strategies may help migraine patients adapt to learning challenges, but further studies are needed to confirm whether neural compensation mechanisms contribute to this adaptation. Cortical compensation, potentially involving the frontal lobe^49^, may play a role in maintaining associative learning abilities in migraine patients. However, future research using neuroimaging and electrophysiological methods will be essential to directly examine the neural basis of these learning patterns.

## Methods

### Participants

Patients were recruited from the Department of Neurology, Albert Szent-Györgyi Health Center, University of Szeged, Hungary, on a voluntary basis without compensation. The inclusion criterion was a diagnosis of migraine without aura, determined by neurologists according to ICHD-3 criteria^53^. Exclusion criteria included any neurological, psychiatric, ophthalmologic, or otologic disorders. Color vision deficiency was assessed using Ishihara plates prior to testing. All testing was conducted during the interictal period, with at least three days having passed since the last migraine attack.

Prior to data collection, we conducted a sample size estimation using α = 0.05, power (1 − β) = 0.80, and an expected effect size of dz = 0.5. This analysis indicated that 35 participants would be required to detect effects of interest with sufficient statistical power. A total of 61 patients were initially recruited for the study. Of these, 41 patients met the inclusion criteria, completed the study per protocol, and were included in the analysis.

The study included 41 migraine patients (34 women and 7 men), aged between 18 and 55 years (mean age 35.3 years, SD 10.9). Participants reported experiencing an average of 3.5 migraine attacks per month, with an estimated lifetime total of 620.9 attacks on average. The average duration of migraine history was 15.3 years (183.5 months), based on the participants’ self-reported onset of headaches. Patients on interval therapy were excluded from the study.

The study protocol adhered to the principles of the Declaration of Helsinki and received approval from the Regional Research Ethics Committee for Medical Research at the University of Szeged, Hungary (27/2020-SZTE).

### Tests and procedures

The tests were conducted on a personal computer equipped with a cathode-ray tube (CRT) screen (refresh rate: 80 Hz). Participants were seated at a comfortable distance from the computer screen. A joystick with left and right buttons was used for recording responses. Testing was conducted individually in a quiet room to minimize distractions and ensure full engagement with the tasks. To reduce carry-over effects, the tests were administered in a pseudorandom sequence. Participants were allowed to complete the tests at their own pace, with no time limits or forced responses to avoid time pressure.

**Fig. 5:**
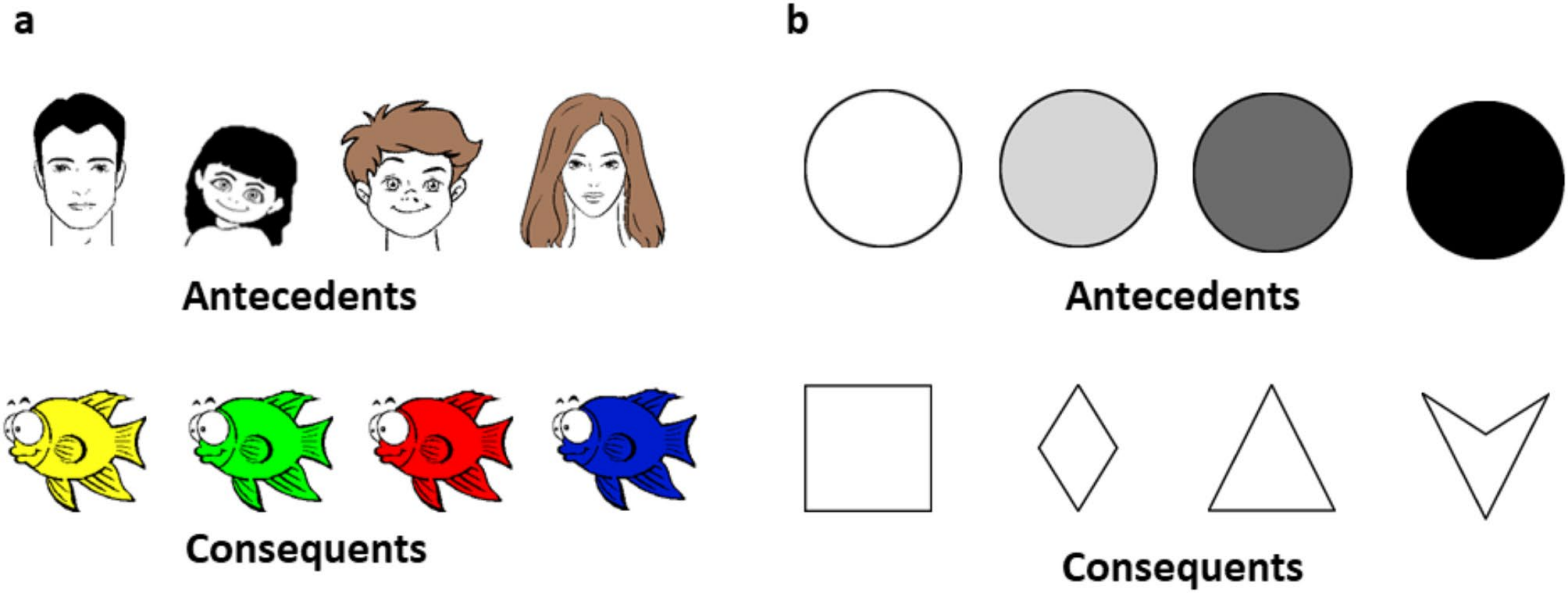
The visual stimulus sets. (**a**) In the RAET test, the antecedents were drawn faces, and the consequents were drawn fish of different colors. (**b**) In the Polygon test, the antecedents were grayscale circles, and the consequents were simple two-dimensional geometric shapes.

The Rutgers Acquired Equivalence Test (RAET)^15^ used in this study was a modified and translated version of the original, authorized by the original authors^11^. The primary objective of the RAET is to learn associations between four antecedents (A1, A2, B1, B2) and four consequents (X1, X2, Y1, Y2) based on feedback. The antecedents consisted of drawn faces (a man, a woman, a boy, and a girl), and the consequents were identical drawn fish in different colors. In contrast, the Polygon test used grayscale circles as antecedents and various two-dimensional blank geometric shapes (square, triangle, rhombus, and concave deltoid) as consequents (Fig. 5).

**Fig. 6:**
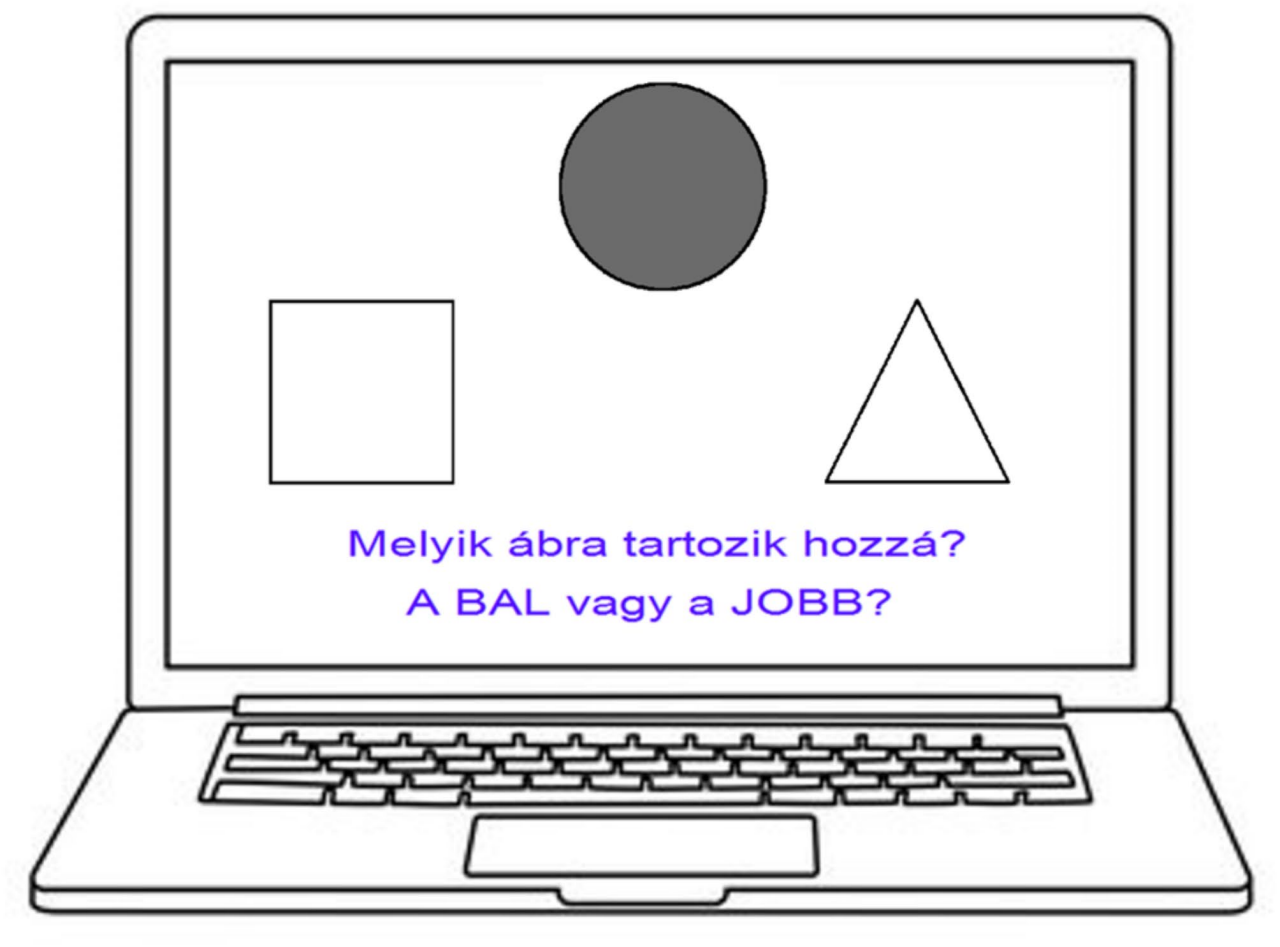
Example of a trial during the acquisition phase of the Polygon test. In each trial, one antecedent is displayed in the center of the screen, with two consequents positioned on the left and right sides. Participants must decide which of the two consequents corresponds to the given antecedent by pressing either the left or right button. The text displayed on the screen translates to: “Which figure belongs to it? The LEFT or the RIGHT?”

The tests consist of two main phases: the acquisition phase and the test phase. During the acquisition phase, participants learn the association pairs through feedback. In each trial, an antecedent (a face or a grayscale circle) is displayed in the middle of the screen, while two consequents (fish or geometric shapes) are shown below it (Fig. 6). Participants must decide which consequent corresponds to the given antecedent by pressing either the left or right button on the joystick.

When an association pair is presented for the first time, the participant’s choice is random. After selecting an option, immediate feedback is provided regarding the correctness of their response: a green check mark accompanied by the word “correct” (“Helyes!“) for correct answers, and a red X accompanied by the word “incorrect” (“Helytelen!“) for incorrect answers. This feedback informs the participant of the correct association.

The association pairs were taught gradually, one pair at a time. After introducing each new stimulus pair, participants were required to achieve a specific number of consecutive correct responses (4, 6, 8, 10, or 12 for each successive new association) to progress and complete the acquisition phase. Consequently, the number of trials in the acquisition phase depended on individual learning performance.

Initially, the first two pairs of stimuli were introduced (A1: X1 and B1: Y1). In the next step, new antecedents were introduced that shared common consequents with the first two pairs (A2: X1 and B2: Y1), resulting in the formation of equivalence between these pairs (A1 = A2 and B1 = B2). In the final step of the acquisition phase, two additional consequents were introduced (A1: X2 and B1: Y2). Of the eight possible associations (stimulus pairs), six were taught to each participant.

In the test phase, participants no longer received feedback about the correctness of their answers. They were required to recall the associations they had already learned (retrieval). Additionally, the application of acquired equivalence to new instances (generalization) was tested by presenting the two remaining pairs (A2: X2 and B2: Y2). If equivalence learning was successful, participants could deduce the correct answers. Participants were not informed in advance about the introduction of these two previously unlearned pairs (generalization).

**Fig. 7:**
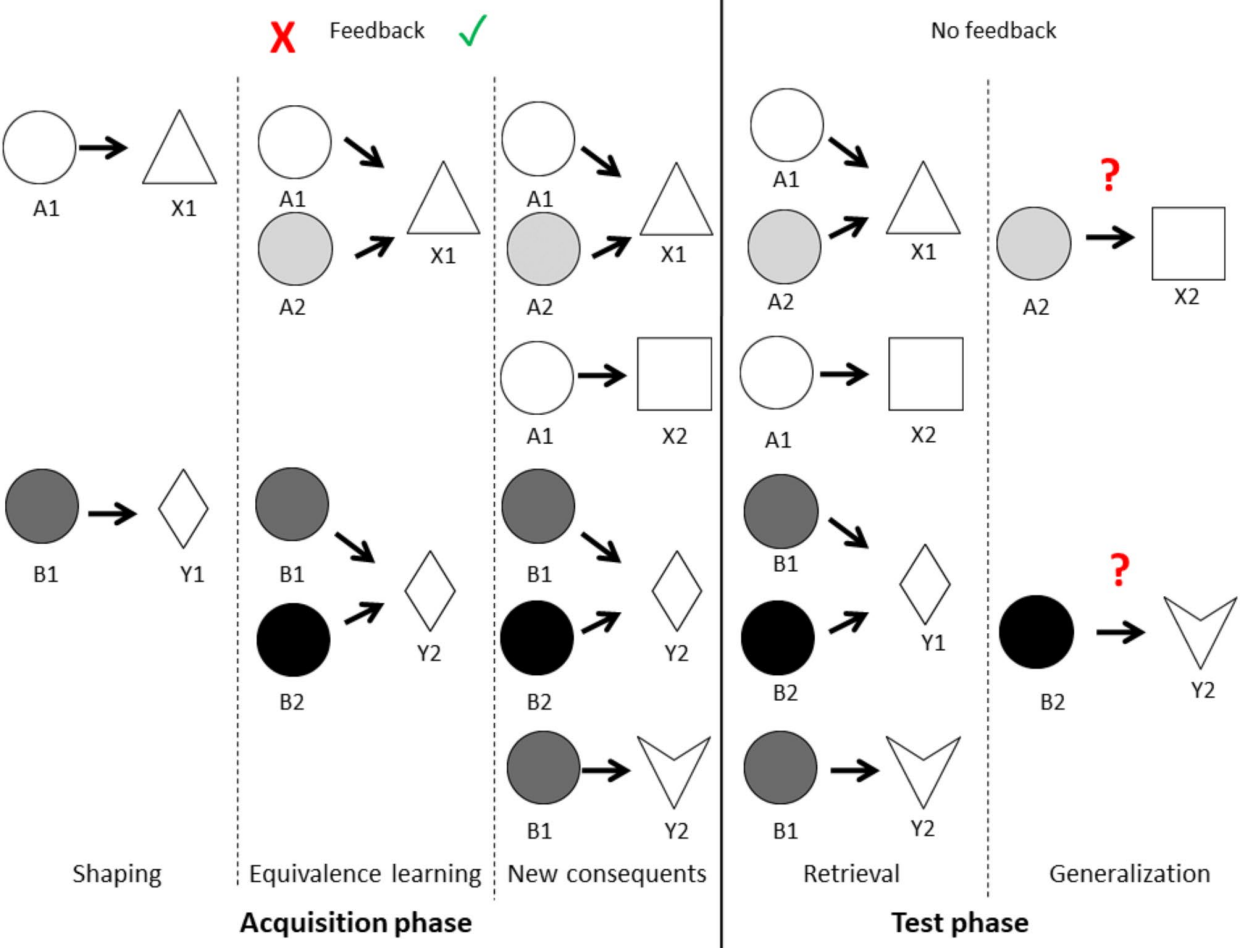
Overview of the test structure as illustrated for the Polygon test. Both tests share the same structure and differ only in the visual stimuli used.

Unlike the acquisition phase, the number of trials in the test phase was fixed, comprising 36 retrieval trials and 12 generalization trials, mixed randomly. Error ratios provided information about performance in both parts of the test phase. The overall structure of the tests is shown in Fig. 7.

### Data analysis

The performances of the participants were evaluated using four parameters: the number of trials needed to learn the associations (NAT), the acquisition learning error ratio (ALER), the retrieval error ratio (RER), and the generalization error ratio (GER). Error ratios were calculated by dividing the number of incorrect answers by the total number of trials for each respective phase. Additionally, reaction times (RT) were measured in milliseconds for each trial, and an average was calculated for each phase (acquisition, retrieval, and generalization). Reaction time was defined as the interval between the appearance of the stimuli and the participant’s response by pressing a button on the joystick. Data points with reaction times exceeding 3 standard deviations (3SD) were excluded from further analysis.

Statistical analysis was performed using Statistica 14.0.0.15 (TIBCO Software Inc.) and G*Power (version 3.1.9.7). The Shapiro-Wilk test was applied to assess the normality of the data sets. As the data did not follow a normal distribution, Wilcoxon Matched-Pairs tests were used to compare performances and reaction times between the RAET and Polygon tests within migraine group. We also conducted a comparison with previously collected healthy data^36^,applying the Mann–Whitney U test to compare both tests between the current migraine group and the healthy group. Effect sizes were calculated using the rank-biserial correlation (r), determined as $\:r=\:Z/\surd\:N$, and eta squared (η^2^), computed as η^2^ = Z^2^/N. Post-hoc power analysis for the Wilcoxon test was conducted in G*Power, with effect size (dZ) calculated as dz = r x 1.25. Power (1 – β) was reported for each comparison to assess the likelihood of detecting an effect given the observed data.

## Data Availability

The datasets generated during and/or analysed during the current study are available from the corresponding author on reasonable request.
